# Impact of preoperative inflammatory biomarkers on postoperative pneumonia and one-month pulmonary imaging changes after surgery for non-small cell lung cancer

**DOI:** 10.3389/fonc.2025.1489068

**Published:** 2025-03-18

**Authors:** Yingding Ruan, Wenjun Cao, Jianwei Han, Aiming Yang, Jincheng Xu, Ting Zhang

**Affiliations:** ^1^ Department of Thoracic Surgery, The First People’s Hospital of Jiande, Jiande, China; ^2^ Department of Thoracic Surgery, Affiliated Zhongshan Hospital of Dalian University, Dalian, China; ^3^ Radiotherapy Department, Second Affiliated Hospital, Zhejiang University School of Medicine, Hangzhou, China

**Keywords:** preoperative inflammatory biomarkers, postoperative pneumonia, chest computed tomography, surgery, non-small cell lung cancer

## Abstract

**Background:**

This study examined the effectiveness of preoperative inflammatory markers in predicting the occurrence of postoperative pneumonia (POP) and clinical outcomes based on chest computed tomography (CT) images in patients who underwent surgical resection for non-small cell lung cancer (NSCLC).

**Methods:**

This retrospective study included NSCLC patients who underwent lung cancer surgery at The First People’s Hospital of Jiande between January 2019 and October 2023. Data on demographic characteristics, preoperative inflammatory biomarkers, surgical approach and duration, postoperative outcomes, and CT findings 1 month postoperatively were collected and analyzed. The effectiveness of preoperative inflammatory markers in predicting POP and clinical outcomes 1 month after surgical resection was assessed using propensity score matching.

**Results:**

Among 568 patients, 72 (12.7%) had POP. After matching, 252 patients (POP group: 66; non-POP group: 186) were included in the analysis. The systemic immune-inflammation index (SII) and platelet-to-lymphocyte ratio (PLR) were significantly higher in the POP group than in the non-POP group (433.53 vs. 323.75, *P* = 0.001; 126.42 vs. 103.64, *P* < 0.001). The length of hospital stay and the percentage of patients who improved clinically based on chest CT findings 1 month after surgery were significantly higher in the POP group than in the non-POP group (11 days vs. 9 days, *P* = 0.008; 77.3% vs. 59.7%, *P* = 0.033). Multivariate analysis showed that PLR and the lymphocyte-to-monocyte ratio (LMR) were independent predictors of POP (AUC of 0.780 and 0.730, both at *P* < 0.001). However, there were no significant differences in postoperative radiographic outcomes among patients stratified by risk of POP.

**Conclusion:**

PLR and LMR accurately predict POP in surgical patients with NSCLC. Nonetheless, these ratios may not significantly predict radiographic outcomes 1 month after surgical resection.

## Introduction

Lung cancer (LC) is the most common malignancy globally, with high morbidity and mortality, imposing a significant health and economic burden (1, 2). Surgery is an effective treatment for patients with LC, especially early-stage non-small cell lung cancer (NSCLC) (3). However, surgical complications, including postoperative pneumonia (POP), may occur, prolonging hospital stay and increasing healthcare costs (4). Despite advancements in antimicrobial therapy and non-invasive respiratory support in the perioperative period, pulmonary infections are a significant cause of mortality in patients with LC (5–8).

The effective prevention and diagnosis of POP are essential components of the surgical treatment of LC. White blood cell (WBC) count, procalcitonin, and C-reactive protein are inflammatory markers of pulmonary infections and have been used to assess the inflammatory status in patients with LC (9–11). The neutrophil-to-lymphocyte ratio (NLR), platelet-to-lymphocyte ratio (PLR), lymphocyte-to-monocyte ratio (LMR), and systemic immune-inflammation index (SII, platelet count × neutrophil count/lymphocyte count) are prognostic factors in LC (12–16). However, the ability of these inflammatory markers to predict the occurrence of POP in surgical patients with LC is unknown.

Our study has important clinical implications. Understanding the role of inflammatory biomarkers in postoperative outcomes can guide personalized perioperative care, potentially reducing the incidence of POP and improving patient recovery. Additionally, the assessment of 1-month pulmonary imaging changes provides a practical and objective measure of postoperative lung function and recovery, which is critical for monitoring patient progress and adjusting treatment plans. This study assessed the clinical value of preoperative inflammatory markers to predict POP and postoperative radiographic outcomes in patients who underwent surgical resection for NSCLC. Elucidating the predictive value of these biomarkers can improve perioperative management and surgical outcomes in patients with NSCLC.

## Patients and methods

### Study population and eligibility criteria

Patients who underwent surgical resection for LC at The First People’s Hospital of Jiande (Jiande, China) from January 2019 to December 2023 were evaluated retrospectively. The inclusion criteria were patients who underwent lung resection and those with a diagnosis of NSCLC. The exclusion criteria were (1) patients with benign disease (n=56); (2) those who underwent repeat surgery (n=10); (3) patients who underwent more than one surgery within 1 month (n=1); (4) those who received radiotherapy, chemotherapy, immunotherapy, targeted therapy, or other treatments before surgery (n=5); (5) patients with second primary lung tumors (n=4); (6) those with infections or autoimmune diseases requiring antibiotic or hormone therapy, including Crohn’s disease and systemic lupus erythematosus (n=31); (7) patients with stage IV NSCLC or received palliative surgery (n=9); and (8) patients transferred to other hospitals (n=1). Blood samples were collected from all patients within 3 days before surgery, and inflammatory markers were measured.

All patients were re-staged according to the eighth edition of the tumor, node, and metastasis (TNM) classification established by the International Association for the Study of LC (17).

This study complied with the Declaration of Helsinki and was approved by the Ethics Committee of The First People’s Hospital of Jiande. The requirement for informed consent was waived because of the retrospective nature of the study.

### Data collection

The following data were collected retrospectively: demographic characteristics (sex, age, body mass index, and smoking history), clinicopathologic features, comorbidities (hypertension, diabetes, coronary heart disease, emphysema, and chronic obstructive pulmonary disease [COPD]), surgical approach, TNM stage, resection site, type of lung resection, number of mediastinal lymph nodes resected and number of nodal stations sampled, surgical duration, intraoperative blood loss volume, drainage time and volume, length of postoperative hospital stay, incidence of POP, histological type of NSCLC, and chest computed tomography (CT) findings 1 month after resection.

The following inflammatory markers were measured to assess inflammatory status preoperatively: neutrophil count, lymphocyte count, macrophage count, platelet count, NLR, PLR, LMR, and SII.

### Observation indicators

This study examined three outcomes. The first outcome was the incidence of POP. The diagnosis of POP was based on the presence of at least three of the following features: (1) lung exudation and consolidation on chest radiographs or CT scans, (2) fever (body temperature > 38°C), (3) WBC count > 10000/mm^3^ or < 3000/mm^3^, (4) opportunistic pathogens in the sputum or bronchial secretions obtained by bronchoscopy (18). The second outcome was the predictive value of preoperative inflammatory markers in POP. The third outcome was clinical outcomes based on CT images obtained immediately after and 1 month after surgery. Two thoracic surgeons (Yingding Ruan and Jianwei Han) classified the outcomes into three categories based on CT findings: worse, unchanged, and improved. Disagreements were resolved by a third investigator (Ting Zhang).

### Statistical analysis

Propensity score matching (PMS) (1:3 ratio) was performed to enhance comparisons and minimize bias. Propensity scores were calculated using a logistic regression model that included gender, age, body mass index, smoking history, surgical approach, TNM stage, resection sites, and type of resection. This approach allowed us to create a cohort that balanced the distribution of these potential confounding factors between the groups.

Additionally, we employed the standardized mean difference (SMD) to assess the balance of these covariates post-matching. Generally, an SMD value less than 0.10 is considered indicative of acceptable balance between groups, with values between 0.10 and 0.34 suggesting minor imbalance, 0.35 to 0.64 indicating moderate imbalance, 0.65 to 1.19 suggesting substantial imbalance, and SMD values of 1.20 or greater indicating a very large imbalance. Our results demonstrated that the SMD values for all covariates were within the acceptable range, confirming good balance between the matched groups.

Normally distributed continuous variables were compared using Student’s t-test and presented as means ± standard deviations. Non-normally distributed continuous variables were compared using the Wilcoxon rank-sum test and expressed as medians and 25th–75th percentiles. Categorical variables were compared using the Chi-square test or Fisher’s exact test and presented as percentages.

Univariate and multivariate analyses were conducted using binary logistic regression models. Variables with a p-value < 0.05 in the univariate analysis were considered significant and were included in the multivariate model to identify independent predictors. The multivariate analysis methods used in the study included backward elimination with a significance level of 0.05 for retention in the model.

Receiver operating characteristic (ROC) curve analysis was performed to determine the optimal cutoff for preoperative inflammatory factors. The area under the curve (AUC) values were calculated, and an AUC ≥ 0.7 was deemed clinically effective. All tests were two-sided, and a p-value of less than 0.05 was considered statistically significant. All statistical analyses were performed using SPSS version 22.0, ensuring the rigorous control of biases and providing detailed insights into the relationships between the study variables.

## Results

### Demographic and baseline characteristics

**Figure 1:**
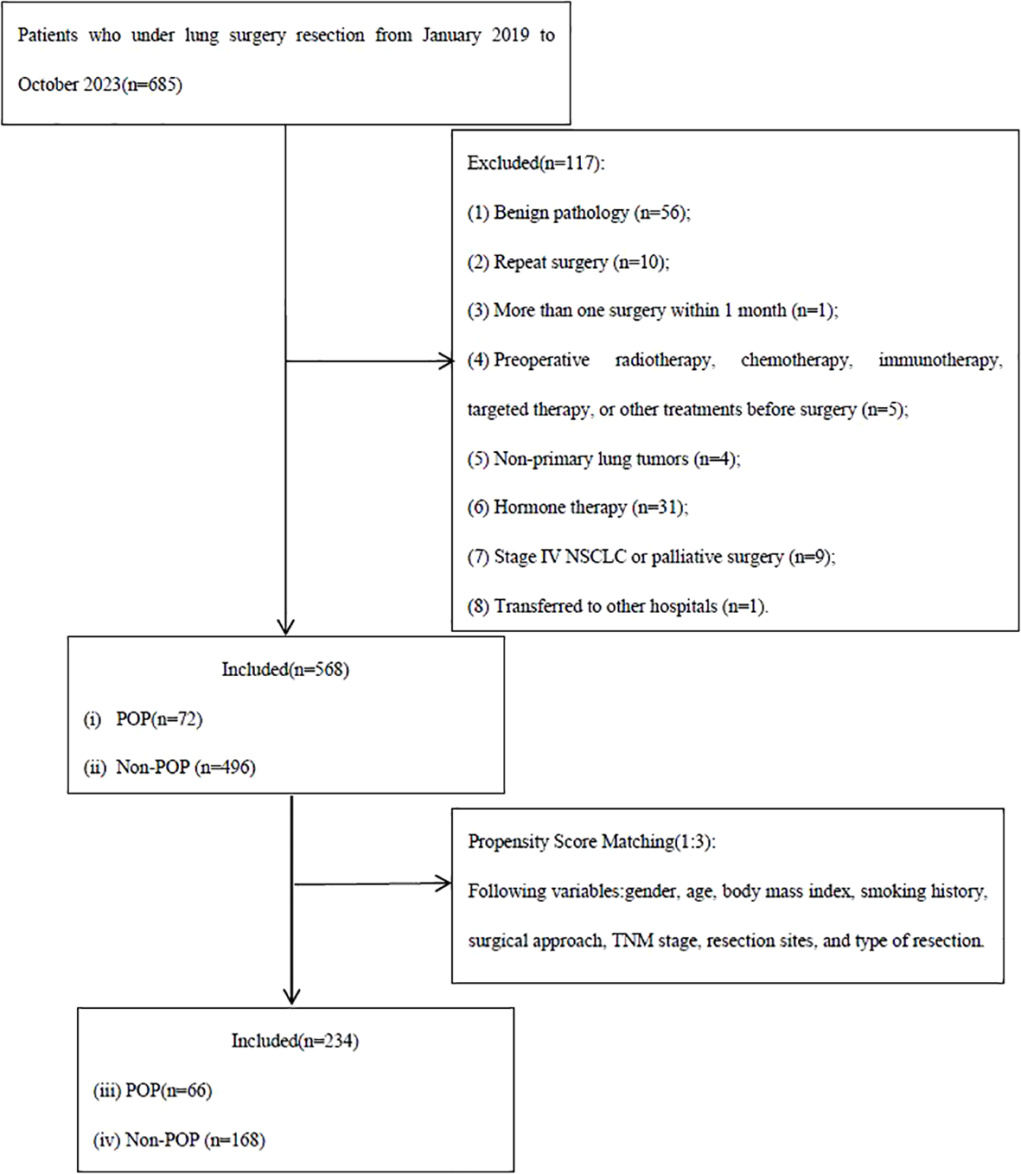
Flowchart of patient selection. POP, postoperative pneumonia.

The study enrolled 685 patients treated surgically for LC in our hospital from January 2019 to December 2023. A total of 568 patients were included in the study after applying the eligibility criteria. After PSM, 252 patients (157 males [62.3%] and 95 females [37.7%]; mean age, 63.89 ± 11.43 years; 66 [26.2%] with POP and 186 [73.8%] without POP) were included in the analysis. The flowchart of patient selection is shown in **Figure 1**. The demographic, clinical, and operative characteristics of the cohort before and after PSM are shown in **Table 1**.

**Table 1:** Demographic, clinical, and operative characteristics of patients with non-small cell lung cancer before and after propensity score matching.

Variables	Before propensity score matching	After propensity score matching
	n = 568	n = 252
Postoperative pneumonia, n (%)	72 (12.7)	66 (26.2)
Sex, n (%)		
Male	292 (51.4)	157 (62.3)
Female	276 (48.6)	95 (37.7)
Smoking, n (%)	187 (32.9)	92 (36.5)
Age (mean ± SD)	62.520 ± 11.098	63.890 ± 11.426
Histological type, n (%)		
Adenocarcinoma	473 (83.3)	205 (81.3)
Squamous cell carcinoma	66 (11.6)	37 (14.7)
Rare NSCLC	29 (5.1)	10 (4.0)
TNM stage, n (%)		
IA or IB	516 (90.8)	213 (84.6)
IIA or IIB	26 (4.6)	25 (10)
IIIA or IIIB	26 (4.6)	14 (5.6)
Body mass index (mean ± SD)	22.861 ± 3.637	23.647 ± 3.857
Intraoperative bleeding volume [M (P25, P75)]	100 (50, 100)	100 (50, 100)
Surgical duration [M (P25, P75)]	126.5 (90, 175)	139.5 (96, 188.25)
Postoperative hospital stay [M (P25, P75)]	8 (5, 11)	10 (7, 13)
Resection site, n (%)		
Right upper	185 (32.6)	58 (23.0)
Right middle	37 (6.5)	47 (18.7)
Right lower	111 (19.5)	15 (6.0)
Left upper	131 (23.1)	79 (31.3)
Left lower	104 (18.3)	53 (21.0)
Type of lung resection, n (%)		
Lobectomy	271 (47.7)	82 (32.5)
Segmental	239 (42.1)	150 (59.5)
Wedge	58 (10.2)	20 (8.0)
Surgical approach, n (%)		
U-VATS	340 (59.9)	125 (49.6)
M-VATS	214 (37.7)	10 (4.0)
Thoracotomy	14 (2.5)	117 (46.4)
Comorbidities, n (%)	265 (46.7)	127 (50.4)
Number of mediastinal lymph nodes resected [M (P25, P75)]	1 (0, 8)	4.5 (0, 10)
Number of stations sampled [M (P25, P75)]	1 (0, 3)	2 (0, 3)
Drainage volume [M (P25, P75)]	800 (450, 1272.5)	975 (600, 1682.5)
Drainage time [M (P25, P75)]	5 (3, 9)	6(4, 10)
Preoperative albumin level (mean ± SD)	43.09 ± 4.813	42.24 ± 4.468
Postoperative albumin level (mean ± SD)	33.90 ± 4.385	33.53 ± 4.539
Antibiotics, n (%)	403 (71.0)	218 (86.5)
Platelets [M (P25, P75)]	180 (143, 221)	178 (143.5, 224.5)
Neutrophils [M (P25, P75)]	3.4 (2.7, 4.5)	3.4 (2.6, 4.5)
Lymphocytes [M (P25, P75)]	1.3 (1.1, 1.7)	1.4 (1.1, 1.7)
Monocytes [M (P25, P75)]	0.3 (0.3, 0.4)	0.3 (0.3, 0.4)
pSII [M (P25, P75)]	338.46 (268.66, 430.96)	336.53 (253.08, 451.48)
PLR [M (P25, P75)]	106.75 (88.31, 131.27)	106.12 (89.76, 129.63)
NLR [M (P25, P75)]	2.64 (1.91, 3.80)	2.720 (1.91, 3.76)
LMR [M (P25, P75)]	3.5 (2.67, 4.30)	3.5 (2.67, 4.33)
Chest CT findings, n (%)		
Improved	376 (66.2)	162 (64.3)
Unchanged	122 (21.5)	60 (23.8)
Worse	70 (12.3)	30 (11.9)

U-VATS, uniportal video-assisted thoracic surgery; M-VATS, multiportal video-assisted thoracic surgery; POP, postoperative pneumonia; pSII, preoperative systemic immune-inflammation index; NLR, neutrophil-to-lymphocyte ratio; PLR, platelet-to-lymphocyte ratio; LMR, lymphocyte-to-monocyte ratio; TNM, tumor, node, and metastasis; M (P25, P75), median (25th–75th percentile); CT, computed tomography; SD, standard deviation.

PSM effectively eliminated confounding factors (**Table 2**). Compared with controls, the POP group exhibited significantly higher preoperative SII values (433.53 [308.49, 606.14] vs. 323.75 [248.98, 404.31], *P* = 0.001) and PLR (126.42 [94.11, 167.10] vs. 103.64 [87.17, 121.64], *P* < 0.001). The LMR was similar between these two groups (3.5 [2.5, 4] vs. 3.5 [2.87, 5], *P* = 0.051). Postoperative hospital stay was considerably longer in the POP group than in the non-POP group (11 days vs. 9 days; *P* = 0.008). The analysis of chest CT scans showed that the percentage of patients who improved after surgery was significantly higher in the POP group than in the control group (77.3% vs. 59.7%, *P* = 0.033).

**Table 2:** Patient characteristics, incidence of POP, and statistical analysis [n(%), mean ± standard deviation, M(P25, P75)].

Variables	Before propensity matching			After propensity matching			
	POP (n=72)	non-POP (n=496)	P-value	POP (n=66)	non-POP (n=186)	P-value	SMD
Sex, n (%)			0.023			0.972	0.005
Male	46 (63.9)	246 (49.6)		41 (62.1)	116 (62.4)		
Female	26 (36.1)	250 (50.4)		25 (37.9)	70 (37.6)		
Smoking, n (%)			0.538			0.977	0.006
Yes	26 (36.1)	161 (32.5)		24 (36.4)	68 (36.6)		
No	46 (63.9)	335 (67.5)		42 (63.6)	118 (63.4)		
Age (mean ± SD)	63.88 ± 11.61	62.32 ± 11.02	0.268	63.61 ± 11.85	64.00 ± 11.30	0.813	0.034
Pathological types, n (%)			0.101			0.535	0.088
Adenocarcinoma	55 (76.4)	418 (84.3)		52 (78.8)	153 (82.3)		
Squamous cell carcinoma	14 (19.4)	52 (10.5)		11 (16.7)	26 (14.0)		
Rare NSCLC	3 (4.2)	26 (5.2)		3 (4.5)	7 (3.7)		
TNM stage, n(%)			0.088			0.642	0.076
IA or IB	67 (92.9)	446 (90.5)		62 (93.7)	160 (86.1)		
IIA or IIB	4 (5.6)	22 (4.4)		3 (4.5)	10 (5.4)		
IIIA or IIIB	1 (1.4)	25 (5.0)		1 (1.5)	16 (8.7)		
Body mass index (mean ± SD)	24.00 ± 4.655	22.70 ± 3.441	0.029	23.770 ± 4.588	23.603 ± 3.576	0.79	0.041
Intraoperative bleeding volume [M (P25, P75)]	100 (50, 100)	50 (50, 100)	0.01	100 (50, 100)	100 (50, 100)	0.184	0.077
Surgical duration [M (P25, P75)]	151.5 (113.8, 189.3)	122.0 (85.0, 171.3)	0.004	141.5 (111.3, 187.3)	137.5 (93.5, 189.3)	0.4	0.077
Postoperative hospital stay [M (P25, P75)]	11 (9, 14)	8 (5, 11)	<0.001	11 (8, 14)	9 (6, 13)	0.008	0.254
Resection Site (n, %)			0.483			0.086	0.303
Right upper	29 (40.3)	156 (31.5)		27 (40.9)	52 (28.0)		
Right middle	3 (4.2)	34 (6.9)		3 (4.5)	12 (6.5)		
Right lower	14 (19.4)	97 (19.6)		12 (18.2)	41 (22.0)		
Left upper	17 (23.6)	114 (22.8)		15 (22.7)	43 (23.1)		
Left lower	9 (12.5)	95 (19.2)		9 (13.6)	38 (20.4)		
Type of lung resection, n (%)			0.012			0.741	0.098
Lobectomy	46 (63.9)	225 (45.4)		40 (60.6)	110 (59.1)		
Segmental	22 (30.6)	217 (43.8)		22 (33.3)	60 (32.3)		
Wedge	4 (5.5)	54 (10.8)		4 (6.1)	16 (8.6)		
Surgical approach, n (%)			0.012			0.8	0.045
U-VATS	33 (45.8)	307 (61.9)		32 (48.5)	93 (50.000)		
M-VATS	35 (48.6)	179 (36.1)		31 (47.0)	86 (46.237)		
Thoracotomy	4 (5.6)	10 (2.0)		3 (4.5)	7 (3.8)		
Comorbidities, n (%)			0.722			0.35	0.134
Yes	35 (48.6)	230 (46.4)		30 (45.5)	97 (52.2)		
No	37 (51.4)	266 (53.6)		36 (54.5)	89 (47.8)		
Number of mediastinal lymph nodes resected [M (P25, P75)]	6 (0, 12)	1 (0, 7)	0.001	6 (0., 10)	4 (0, 10)	0.949	0.028
Number of stations sampled [M (P25, P75)]	2 (0, 3)	1(0, 3)	0.004	2 (0, 3)	2 (0, 3)	0.642	0.051
Drainage volume [M (P25, P75)]	1072.5 (657.5, 1705.0)	750.0 (421.5, 1212.5)	<0.001	1037.5 (650.0, 1685.0)	950.0 (550.0, 1660.0)	0.344	0.027
Drainage time [M (P25, P75)]	7 (4, 11)	5 (3, 8)	<0.001	7 (4, 11)	6 (4, 10)	0.436	0.022
Preoperative albumin level (mean ± SD)	41.79 ± 5.15	43.28 ± 4.74	0.014	42.19 ± 4.82	42.27 ± 4.35	0.903	0.017
Postoperative albumin level (mean ± SD)	33.42 ± 4.5095	34.00 ± 4.35	0.322	33.54 ± 4.43	33.52 ± 4.59	0.984	0.003
Antibiotics (%)			<0.001			0.704	0.055
Yes	64 (88.9)	339 (68.3)		58 (87.9)	160 (86.0)		
No	8 (11.1)	157 (31.7)		8 (12.1)	26 (14.0)		
Platelets [M (P25, P75)]	180 (144.75, 22)	173.5 (133, 24)	0.681	173.5 (133, 234.25)	173 (131.2, 237.5)	0.339	0.010
Neutrophils [M (P25, P75)]	3.4 (2.9, 4.3)	3.4 (2.7, 4.5)	0.619	3.4 (2.9, 4.4)	3.3 (2.6, 4.6)	0.782	0.019
Lymphocytes [M (P25, P75)]	1.3 (1.1, 1.7)	1.3 (1.1, 1.8)	0.71	1.4 (1.1, 1.7)	1.3 (1.0, 1.8)	0.531	0.008
Monocytes [M (P25, P75)]	0.3 (0.3, 0.4)	0.4 (0.3, 0.5)	0.116	0.3 (0.3, 0.5)	0.3 (0.3, 0.4)	0.691	0.004
pSII [M (P25, P75)]	433.53 (306.61, 603.19)	334.17 (267.50, 409.64)	<0.001	433.53 (308.49, 606.14)	323.75 (248.98, 404.31)	0.001	0.460
PLR [M (P25, P75)]	126.97 (94.33, 165.03)	105.13 (87.97, 128.33)	<0.001	126.42 (94.11, 167.10)	103.64 (87.17, 121.64)	<0.001	0.557
NLR [M (P25, P75)]	2.61 (1.94, 3.34)	2.67 (1.88, 3.89)	0.719	2.63 (1.94, 3.32)	2.76 (1.87, 3.98)	0.557	0.027
LMR [M (P25, P75)]	3.5 (2.7, 4.3)	3.6 (3.0, 5.0)	0.037	3.5 (2.5, 4)	3.5 (2.87, 5)	0.051	0.364
Chest CT radiological findings, n (%)			0.06			0.009	0.097
Improved	56 (77.8)	320 (64.5)		51 (77.3)	111 (59.7)		
Unchanged	12 (16.7)	110 (22.2)		11 (16.7)	49 (26.3)		
Worse	4 (5.5)	66 (13.3)		4 (6.0)	26 (14.0)		

U-VATS, uniportal video-assisted thoracic surgery; M-VATS, multiportal video-assisted thoracic surgery; POP, postoperative pneumonia; pSII, preoperative systemic immune-inflammation index; NLR, neutrophil-to-lymphocyte ratio; PLR, platelet-to-lymphocyte ratio; LMR, lymphocyte-to-monocyte ratio; TNM, tumor, node, and metastasis; M (P25, P75), median (25th–75th percentile); CT, computed tomography; SD, standard deviation; SMD, standardized mean difference.

### Risk factors for POP in NSCLC

After PSM, clinical data were analyzed by univariate and multivariate logistic regression analysis. Univariate analysis revealed that SII, PLR, LMR, and preoperative chest CT findings were significant risk factors for POP (*P* = 0.002, *P* < 0.001, *P* = 0.008, and *P* = 0.013). Multivariate analysis showed that PLR (Exponential(B) [Exp(B)] = 0.988, 95% confidence interval [CI] = 0.980–0.996, *P* = 0.005) and LMR (Exp(B) = 0.618, 95% CI = 0.494–0.772, *P* < 0.001) were significant predictors of POP (**Tables 3**, **4**).

**Table 3:** Univariate logistic regression analysis of risk factors for postoperative pneumonia in patients with non-small cell lung cancer.

Variables	B	SE	Wald	P	Exp(B)	95% Exp(B) CI	
						Down	Up
Sex	-0.01	0.295	0.001	0.972	0.99	0.555	1.766
Age	0.003	0.012	0.057	0.812	1.003	0.979	1.028
Pathological types	-0.161	0.274	0.345	0.557	0.852	0.498	1.456
TNM stage	0.069	0.089	0.597	0.44	1.071	0.9	1.275
Body mass index	-0.011	0.037	0.091	0.763	0.989	0.92	1.063
Intraoperative bleeding volume	0	0.001	0.293	0.588	1.000	0.999	1.001
Surgical duration	-0.001	0.002	0.265	0.607	0.999	0.994	1.003
Postoperative hospital stay	-0.04	0.024	2.791	0.095	0.96	0.916	1.007
Resection site	0.167	0.096	3.048	0.081	1.182	0.98	1.426
Type of lung resection	0.1	0.228	0.191	0.662	1.105	0.707	1.727
Surgical approach	-0.07	0.249	0.078	0.779	0.933	0.572	1.52
Comorbidities	-0.268	0.288	0.872	0.351	0.765	0.435	1.343
Number of Mediastinal lymph nodes resected	-0.004	0.021	0.040	0.842	0.996	0.956	1.037
Number of stations sampled	-0.026	0.074	0.126	0.723	0.974	0.842	1.127
Drainage volume	0	0	0.03	0.861	1.000	1.000	1.000
Drainage time	0.003	0.023	0.020	0.887	1.003	0.959	1.050
Platelets	0.002	0.002	0.745	0.388	1.002	.997	1.007
Neutrophils	0.030	0.099	0.093	0.760	1.031	0.849	1.251
Lymphocytes	0.129	0.278	0.217	0.642	1.138	0.660	1.964
Monocytes	-0.083	0.935	0.008	0.930	0.921	0.147	5.758
pSII	-0.002	0.001	9.897	0.002	0.998	0.997	0.999
PLR	-0.011	0.003	13.358	<0.001	0.989	0.983	0.995
NLR	0.002	0.011	0.040	0.841	1.002	0.981	1.024
LMR	-0.261	0.099	6.941	0.008	0.770	0.634	0.935
Preoperative albumin level	0.004	0.032	0.015	0.903	1.004	0.943	1.069
Postoperative albumin level	-0.001	0.032	0	0.984	0.999	0.939	1.063
Smoking	0.008	0.298	0.001	0.977	1.008	0.563	1.808
Antibiotics	0.164	0.432	0.144	0.705	1.178	0.505	2.749
Chest CT radiological findings	0.608	0.244	6.21	0.013	1.836	1.139	2.962

POP, postoperative pneumonia; pSII, preoperative systemic immune-inflammation index; NLR, neutrophil-to-lymphocyte ratio; PLR, platelet-to-lymphocyte ratio; LMR, lymphocyte-to-monocyte ratio; TNM, tumor, node, and metastasis; M (P25, P75), median (25th–75th percentile); CT, computed tomography; SD, standard deviation.

**Table 4:** Multivariate logistic regression analysis of risk factors for postoperative pneumonia in patients with non-small cell lung cancer.

Variables	B	SE	Wald	P	Exp(B)	95% Exp(B) CI	
						Down	Up
pSII	-0.001	0.001	2.083	0.149	0.999	0.997	1.000
PLR	-0.012	0.004	7.854	0.005	0.988	0.980	0.996
LMR	-0.482	0.114	17.990	<0.001	0.618	0.494	0.772

pSII, preoperative systemic immune-inflammation index; PLR, platelet-to-lymphocyte ratio; LMR, lymphocyte-to-monocyte ratio.

### Effectiveness of PLR and LMR in predicting POP

**Figure 2:**
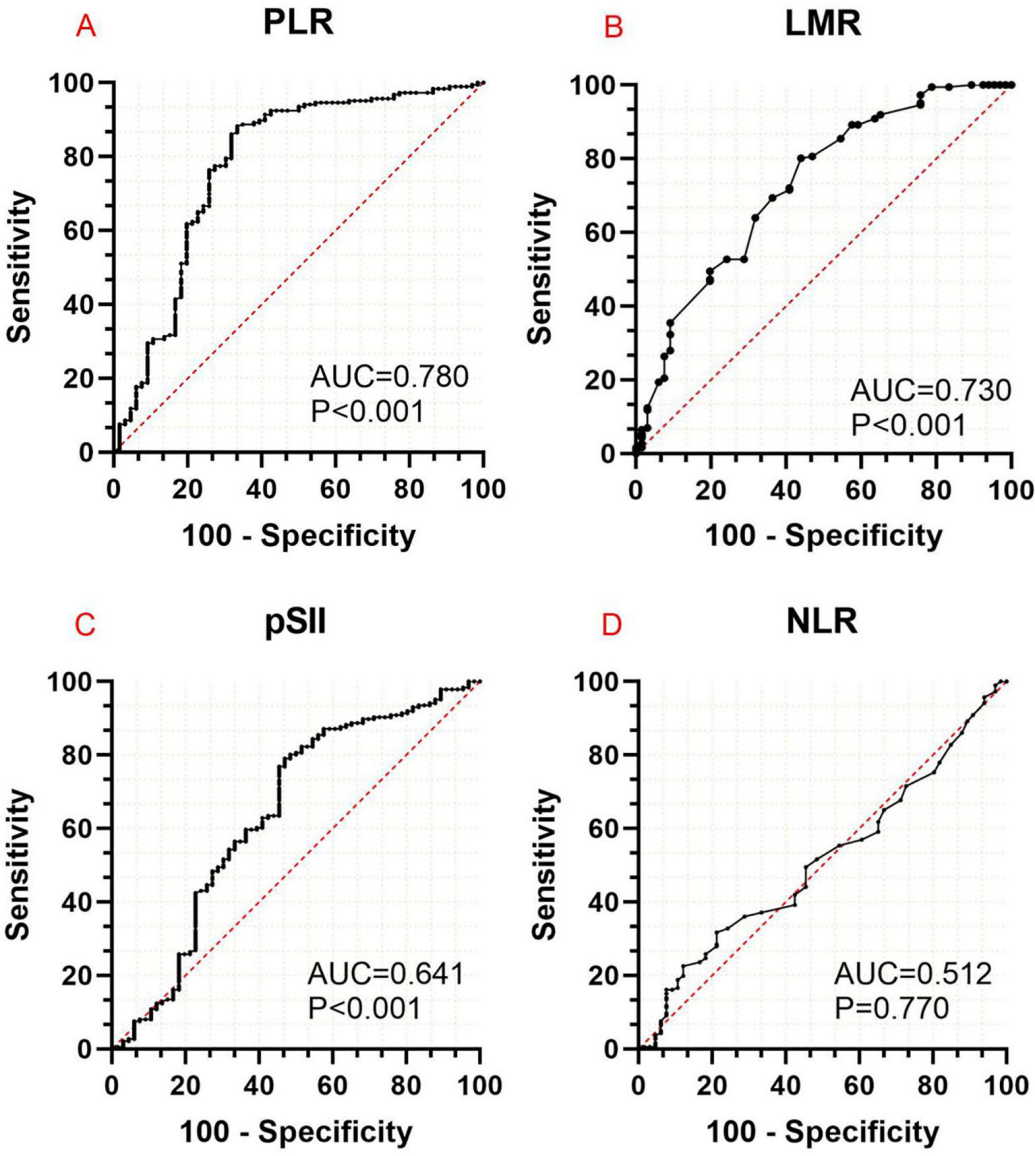
Area under the curve analysis of the effectiveness of PLR **(A)**, LMR **(B)**, SII **(C)**, and NLR **(D)** in predicting the occurrence of postoperative pneumonia in patients with non-small cell lung cancer. PLR, platelet-to-lymphocyte ratio; LMP, lymphocyte-to-monocyte ratio; pSII, preoperative systemic immune-inflammation index; NLR, neutrophil-to-lymphocyte ratio.

AUC analysis showed that PLR and LMR had a higher ability to predict POP (AUC of 0.780 and 0.729, both at *P* < 0.001). The optimal thresholds for PLR and LMR were 140.315 and 4.875. Youden index, sensitivity, and specificity were 0.549, 66.7%, and 88.2% for PLR and 0.362, 56.1%, and 80.1% for LMR. The AUC of preoperative NLR and SII were 0.488 and 0.641 (**Figure 2**).

### Subgroup analysis of patients with POP

PSM analysis showed that PLR and LMR could accurately predict the occurrence of POP in patients with NSCLC. The optimal cutoff values for PLR and LMR based on AUC analysis were 140.315 and 4.875, respectively. The patients were divided into three risk groups based on threshold values: high (PLR > 140.315 and LMR > 4.875), intermediate (PLR > 140.315 or LMR > 4.875), and low (PLR < 140.315 and LMR < 4.875). Postoperative clinical outcomes in each group were classified into three categories—worse, unchanged, and improved—based on the analysis of CT images obtained immediately after and 1 month after surgery. There were no significant differences in outcomes among these groups (*P*= 0.976) (**Table 5**).

**Table 5:** Baseline, clinical, and operative characteristics of non-small cell lung cancer patients stratified into three groups according to the risk of POP.

Variables	Groups according to the risk of POP			P-value
	High (*n* = 6)	Intermediate (*n* = 39)	Low (*n* = 21)	
Sex, n (%)				0.296
Male	3 (50)	22 (56.4)	16 (76.2)	
Female	3 (50)	17 (43.6)	5 (23.8)	
Age (mean ± SD)	63.50 ± 10.464	62.77 ± 11.690	65.19 ± 12.852	0.757
Pathological types, n (%)				0.180
Adenocarcinoma	6 (100)	32 (82.1)	14 (66.7)	
Squamous cell carcinoma	0 (0.0)	5 (12.8)	6 (28.6)	
Rare NSCLC	0 (0.0)	2 (5.1)	1 (4.7)	
TNM stage, n (%)				0.172
IA or IB	2 (100)	36 (92.2)	20 (95.2)	
IIA or IIB	0 (0.0)	2 (5.2)	1 (4.8)	
IIIA	0 (0.0)	1 (2.6)	0 (0.0)	
Body mass index (mean ± SD)	22.48 ± 3.814	23.84 ± 4.877	24.01 ± 4.359	0.770
Intraoperative bleeding volume [M (P25, P75)]	75 (50, 100)	100 (50, 100)	100 (50, 100)	0.437
Surgical duration [M (P25, P75)]	124 (97, 151)	160 (116, 192)	141 (108, 160)	0.382
Postoperative hospital stay [M (P25, P75)]	13 (11, 16.5)	11 (9, 14)	10 (8, 12)	0.218
Resection site, n (%)				0.368
Right upper	4 (66.7)	15 (38.5)	8 (38.1)	
Right middle	0 (0.0)	2 (5.1)	1 (4.8)	
Right lower	1 (16.7)	8 (20.5)	3 (14.3)	
Left upper	1 (16.6)	9 (23.1)	5 (23.8)	
Left lower	0 (0.0)	5 (12.8)	4 (19.0)	
Type of lung resection, n (%)				0.596
Lobectomy	4 (66.7)	25 (64.1)	11 (52.4)	
Segmental	1 (16.7)	13 (33.3)	8 (38.1)	
Wedge	1 (16.7)	1 (2.6)	2 (9.5)	
Surgical approach, n (%)				0.633
U-VATS	2 (33.3)	21 (53.8)	9 (42.9)	
M-VATS	4 (66.7)	16 (41.0)	11 (52.4)	
Thoracotomy	0 (0.0)	2 (5.1)	1 (4.7)	
Comorbidities, n (%)				0.605
Yes	4 (66.7)	17 (43.6)	9 (42.9)	
No	2 (33.3)	22 (56.4)	12 (57.1)	
Number of mediastinal lymph nodes resected [M (P25, P75)]	5 (0, 10)	6 (0, 9)	5 (0, 13)	0.823
Number of stations sampled [M (P25, P75)]	2 (0, 4)	2 (0, 3)	3 (0, 3)	0.836
Drainage volume [M (P25, P75)]	830 (615, 1202)	1190 (635, 1760)	1020 (730, 1550)	0.668
Drainage time [M (P25, P75)]	10 (4, 11)	9 (4, 12)	5 (4, 7)	0.142
Preoperative albumin level (mean ± SD)	43.17 ± 4.63	42.39 ± 5.30	41.52 ± 3.99	0.705
Postoperative albumin level (mean ± SD)	33.73 ± 3.83	33.36 ± 4.81	33.81 ± 4.01	0.926
Smoking, n (%)				0.548
Yes	1 (16.7)	14 (35.9)	9 (42.9)	
No	5 (83.3)	25 (64.1)	12 (57.1)	
Antibiotics, n (%)				0.621
Yes	5 (83.3)	35 (89.7)	18 (85.7)	
No	1 (16.7)	4 (10.3)	3 (14.3)	
Chest CT radiological findings, n (%)				0.976
Improved	5 (83.3)	30 (76.9)	16 (76.2)	
Unchanged	0 (0.0)	6 (15.4)	5 (23.8)	
Worse	1 (16.7)	3 (7.7)	0 (0.0)	

U-VATS, uniportal video-assisted thoracic surgery; M-VATS, multiportal video-assisted thoracic surgery; NSCLC, non-small cell lung cancer; POP, postoperative pneumonia; pSII, preoperative systemic immune-inflammation index; TNM, tumor, node, and metastasis; M (P25, P75), median (25th–75th percentile); CT, computed tomography; SD, standard deviation.

## Discussion

Advancements in video-assisted thoracic surgery, surgical equipment, and perioperative management have increased awareness of the benefits of minimally invasive surgery for LC. However, postoperative complications, especially pulmonary infections, can limit clinical recovery. Thus, accurately predicting these complications is crucial to improve prognosis and treatment. This study investigated the relationship between preoperative inflammatory markers (NLR, LMR, SII, and PLR) and POP using PSM. Further, this study is the first to evaluate POP, inflammatory markers, and 1-month radiographic outcomes in patients with NSCLC. The results revealed that PLR and LMR were independent risk factors for POP, with good predictive value, demonstrating the potential clinical utility of these markers in predicting the occurrence of postoperative pulmonary infections. Additionally, clinical outcomes improved 1 month after surgery in patients with POP based on the analysis of chest CT images, independent of PLR and LMR levels. Consequently, calculating PLR and LMR values in surgical patients with LC upon admission can help identify individuals who can benefit from intensive respiratory support and interventions targeting respiratory infections, improving prognosis (19, 20).

Peripheral blood inflammatory markers, including neutrophils, lymphocytes, macrophages, platelets, and natural killer cells, reflect systemic inflammation and have multiple roles in cancer (21, 22). In the early postoperative period, the decreased level and function of lymphocytes and NK cells can impair cellular immunity, increasing the risk of postoperative pneumonia and other inflammatory diseases (23). However, our results showed that preoperative neutrophils, lymphocytes, platelets, and monocytes had no significant impact on POP. In clinical practice, changes in WBC count may alter NLR, LMR, SII, and PLR. These ratios can reflect systemic and local inflammation and predict the occurrence of postoperative complications (12–14).

Our findings suggest that hematological ratios such as PLR and LMR can better predict the risk of POP. PLR, as a marker of platelet activation and systemic inflammation, has good potential to predict the risk of clinical deterioration in patients with pulmonary diseases (24, 25). Platelets play a crucial role in inflammation by releasing inflammatory mediators such as platelet-derived growth factor and thromboxane A2, which promote inflammatory responses (26, 27). Platelets are activated in response to infection and injury and aggregate at the site of damage, forming thrombi and initiating inflammatory reactions. Lymphocytes, as the central regulators of the immune system, are depleted by stress responses. Therefore, elevated PLR may reflect high-grade systemic inflammation, which may promote the occurrence of POP (28–30). Furthermore, platelets interact with immune cells such as lymphocytes and monocytes, influencing their function and activity. Increased PLR may indicate abnormal interactions of platelets with immune cells, potentially disrupting immune responses and increasing the risk of POP (31).

LMR reflects the balance between lymphocytes and monocytes, which have distinct roles in the immune system. Lymphocytes provide antitumor and anti-infectious defenses (32–34). Monocytes are involved in innate immunity and inflammation, killing pathogens by phagocytosis and the release of inflammatory mediators (35, 36). Decreased LMR may indicate lymphocyte depletion or monocyte expansion, compromising immune function and increasing the risk of infections. In POP, lower LMR may indicate weakened immune defense, rendering the lungs more susceptible to infections and inflammatory insults (37, 38). LMR correlates with disease incidence and prognosis (39–42). LMR is an independent predictor of stroke-associated pneumonia in patients with acute ischemic stroke, particularly when LMR was less than 4 (39).

In our study, LMR values were similar in the POP and non-POP groups (3.5 [2.5, 4] vs. 3.5 [2.87, 5], *P*=0.051), with a cutoff value of 4.875, consistent with a previous study indicating the potential of LMR to predict inflammatory conditions (38). These observations underscore the importance of considering hematological ratios, particularly PLR and LMR, in assessing POP risk and guiding clinical practice.

However, little is known about the ability of NLR, LMR, SII, and PLR to predict the occurrence of POP in surgical patients with LC. A retrospective study involving 1486 patients who underwent LC surgery found that preoperative SII was an independent risk factor for POP and predicted its occurrence (18). SII is also a risk factor for sepsis after intestinal obstruction surgery and for pulmonary complications after LC resection (22, 43). In contrast, NLR had a higher predictive value for POP than PLR and SII in older patients with hip fractures, even after PSM (44). NLR had the highest predictive value for POP in our cohort (AUC = 0.648, 95% CI = 0.594–0.701), maintaining significance even after PSM (Exp(B) = 2.04, 95% CI = 1.31–3.20). These data demonstrate that the impact of preoperative inflammation on POP varies in each study, highlighting the need to clarify this relationship.

In our study, both before and after PSM, PLR and LMR were significantly higher in the POP group than in the non-POP group (before PSM: *P* < 0.001 for PLR and *P* = 0.037 for LMR; after PSM: *P* < 0.001 for PLR and *P* = 0.051 for LMR). Multivariable analysis after PSM showed that PLR (*P* = 0.005) and LMR (*P* < 0.001) were independent risk factors for POP. Further, AUC analysis showed that PLR (ACU = 0.780, *P* < 0.001) and LMR (ACU = 0.730, *P* < 0.001) were better predictors of the occurrence of POP than SII and NLR.

Inflammatory burden, PLR, NLR, and other systemic inflammation markers are good prognostic factors in LC (33, 34, 45–47). However, little is known about the ability of these markers to predict postoperative outcomes in patients with pneumonia. To address this gap, we analyzed the effectiveness of PLR and LMR in predicting radiological outcomes 1 month after surgery. In the total cohort, the proportion of patients with improved outcomes before and after PSM was 66.2% (376/568) and 64.3% (162/252), respectively. In the POP group, the percentage of patients with improved outcomes before and after PSM was 77.8% (56/72) and 77.3% (51/66), respectively. The risk of POP was classified into three categories—low, intermediate, and high—based on the optimal cutoff values of PLR and LMR. There were no significant differences in postoperative radiological outcomes among the three risk groups. This result suggests that while PLR and LMR are good predictors of the risk of POP in patients with NSCLC, their predictive value for short-term radiological outcomes is limited.

Although we demonstrated the relationship of preoperative inflammatory biomarkers with POP and changes in lung imaging 1 month postoperatively, this study acknowledges the importance of other potential confounding factors. Moreover, despite employing PSM to minimize the influence of these factors, the roles of confounding variables such as age, gender, body mass index (BMI), smoking history, COPD status, and comorbidities (diabetes, cardiovascular disease, and tuberculosis) must be considered. For instance, the impact of age on the risk of postoperative pneumonia is controversial. Some studies suggest that immune dysfunction in elderly patients increases the risk of POP (48, 49), while others have shown that age is not an independent risk factor (50). BMI may also be a risk factor for POP (49, 51). For instance, BMI ≥ 24.0 kg/m^2^ was an independent risk factor for POP (51). However, the direct association between BMI and POP has not been demonstrated (48, 50, 52, 53). Smoking and COPD impair lung function and increase the risk of inflammatory responses, while comorbidities may exacerbate inflammatory reactions and affect postoperative recovery (48, 50, 52, 54). Therefore, we considered the effects of these confounding factors to increase the accuracy and reliability of our conclusions.

Surgical approaches, including open surgery and video-assisted thoracoscopic surgery (VATS), may influence the occurrence of POP. For instance, VATS, due to its minimal trauma and rapid recovery, can reduce the risk of POP (55–59). Although this study did not compare these surgical approaches, these findings are important for clinical practice, suggesting that VATS should be considered the preferred surgical option for NSCLC. However, the impacts of different surgical approaches on inflammatory biomarkers and postoperative outcomes require further investigation to improve surgical strategies and patient prognosis.

To prevent POP, we implemented comprehensive management measures, including routine lung rehabilitation training, bronchodilator and steroid therapy for high-risk patients, and anti-inflammatory treatment. In subsequent treatment phases, these measures should be more aggressively implemented for high-risk patients. These approaches have been validated and can significantly reduce the risk of POP (60–62).

This study has limitations. First, the retrospective design may lead to selection bias. Second, data were obtained from hospital records, limiting the effectiveness and reliability of data collection. Notably, we were unable to collect data on patients’ pulmonary function, which could be an important confounder affecting the outcomes of interest. Although biases were reduced using PSM, the small sample size may have limited statistical power. Third, the impact of inflammatory markers on long-term patient survival was not analyzed. Fourth, although PLR and LMR could accurately predict POP, diagnostic sensitivity was low (66.7% and 56.1%, respectively), possibly because of the small sample size, patient specificity, or analytical bias. Thus, large-scale, multicenter, prospective studies are needed to confirm the reliability and clinical value of these markers to predict POP in patients with LC.

Despite these limitations, this study evaluated short-term clinical outcomes in patients who developed pneumonia after lung resection for NSCLC. Moreover, the results provide a basis for using preoperative inflammatory markers to improve treatment outcomes and the postoperative recovery of patients with LC.

In conclusion, preoperative PLR and LMR can predict the occurrence of POP in surgical patients with NSCLC. However, PLR and LMR may not predict radiographic outcomes 1 month after surgical resection.

## Data Availability

The original contributions presented in the study are included in the article/supplementary material. Further inquiries can be directed to the corresponding author.
